# Large outbreak of herpangina in children caused by enterovirus in summer of 2015 in Hangzhou, China

**DOI:** 10.1038/srep35388

**Published:** 2016-10-18

**Authors:** Wei Li, Hui-hui Gao, Qiong Zhang, Yu-jie Liu, Ran Tao, Yu-ping Cheng, Qiang Shu, Shi-qiang Shang

**Affiliations:** 1 Department of Clinical Laboratory, Children’s Hospital of Zhejiang University School of Medicine, Hangzhou 310013, PR China

## Abstract

Herpangina, usually caused by coxsackie virus A, is prevalent in children spreading through the fecal-oral transmission and the respiratory droplets dissemination. Also, it is mostly asymptomatic and self-limiting. In our study, we found that large outbreak of herpangina in children occurred in the summer of 2015 in Hangzhou, China. From May 1th to August 31th, a total of 10 210 children were diagnosed with herpangina in Children’s Hospital of Zhejiang University School of Medicine. 2 310 throat swabs were collected and tested for enterovirus detection by real-time RT-PCR, while 1 651 cases were positive with the rate of 71.5%. Based on VP1 gene or 5′UTR region sequences, Coxsackievirus A2, A4, A6, A10, B2, B4 and echovirus 30 were detected in these cases. More importantly, Coxsackievirus A2 may be the major subtype of enterovirus resulting in children with herpangina in hangzhou, China.

Enteroviruses (EVs), belonging to the picornaviridae family, include coxsackievirus A (CVA) and coxsackievirus B (CVB), echoviruses, polioviruses and the numbered enteroviruses^1^,^2^. EVs is one of the most common viruses that causes diseases in human^3^. Particularly, it is the major pathogen thatcauses herpangina, hand-foot-mouth disease (HFMD) and neurological diseases in children throughout the world^4^,^5^,^6^. A large outbreak of HFMD occurred in 2008 in China, with EV71, CA16, and CVA6 identified as the major pathogens causing HFMD^7^,^8^,^9^. Interestingly, in our previous study, we found EV71 and CA16 were decreasing after 2010, while other types of EVs caused herpangina were increasing in Hangzhou^5^. Herpangina, generally considered as one of the asymptomatic and self-limiting infectious diseases in patients with mild clinical complications, can be curedcompletely within 5–7 days after infection^10^. Outbreaks of herpangina were reported in many cities, what’s more, many subtypes of EVs were identified as the pathogens causing herpangina, such as CVA, CVB, and echovirus^10^,^11^,^12^. In this article, we displayed a large outbreak of herpangina in children in the summer of 2015 in Hangzhou, China.

## Results

### Patients’ characteristics

From May 1, 2015 to August 31, 2015, 10 210 patients were diagnosed with herpangina at outpatient department in our hospital. In temporal distribution, the herpangina patients comprised 1 433 children (14.0%) in May, 5 257 children (51.5%) in June, 2 871 children (28.1%) in July, and 649 children (6.4%) in August.

Children enrolled in this study were with a median age of 1.75 years old (ranging from 0.03 to 13.5 years old) at the onset of herpangina and a male to female ratio of 1.26. 9 352 out of 10 210 (91.6%) children were less than 5 years, with constitution ratios of 23.2%, 46.6%, 21.8%, 6.7% and 1.7% in children aged 0-1year, 1–3 years, 3–5 years, 5–9 years and >9 years, respectively.

All children with herpangina were divided into three groups by age and performed with routine blood test and C-reactive protein (CRP) assay. Our results showed that the median number of white blood cells were 7.4 × 10^9^/L, 8.9 × 10^9^/L, 8.2 × 10^9^/L, the median percentage of lymphocyte were 46.4%, 38.2%, 30.3%, the median percentage of neutrophil were 58.5%, 54.3%, 63.2%, and the median level of CRP were 25.1 mg/ml, 15.3 mg/ml, 14.8 mg/ml in each age group (Table. 1).

### Results of enterovirus assay

**Figure 1:**
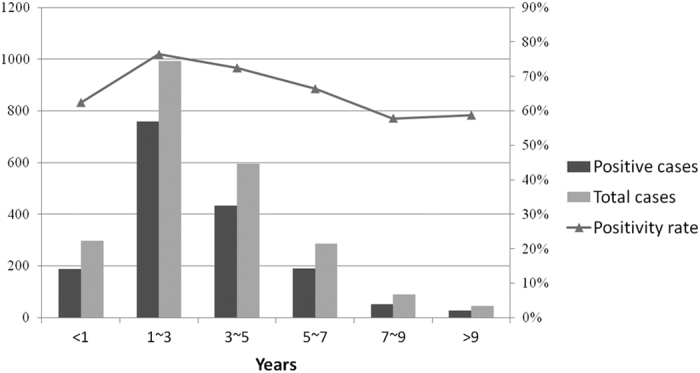
Age distribution of entervirus infections among children with herpangina.

To confirm the major pathogen causing herpangina, 2 310 throat swabs were randomly collected from patients and tested by enterovirus one-step real-time RT–PCR. As a result, 1 651 samples were tested positive for enterovirus with the rate of 71.5%. Positive rate for enterovirus was the highest among children aged 1–3 years (76.4%), and steadily declined with age increase or decrease (Fig. 1). However, children in other age groups, the detection rates of enterovirus infection were still as high as 62.4%, 72.5%, 66.4%,57.8% and 58.7% in <1 year, 3–5 years, 5–7 years, 7–9 years, and >9 years, respectively.

### VP1 gene based phylogenetic analysis

To confirm the subtypes of enterovirus, 35 clinical strains were randomly collected and the VP1 and 5′UTR gene of the EV were amplified by the conventional RT-PCR (Figure S1). The amplification products were purified, sequenced and then used for phylogenetic analysis. After comparing the VP1 and 5′UTR genes withdifferent referenced EV strains, the sequence homology was displayed as 90.9–99.5% (nucleotide), which met the serotype identification criteria for homologous serotypes. The molecular typing results showed that the major EV isolates belonged to the human EVA and EVB species, including 22 samples with Coxsackievirus A2, 3 samples with Coxsackievirus A10, 1 sample with Coxsackievirus A6, 2 samples with Coxsackievirus B2, 1 sample with Coxsackievirus B4 and 3 samples with Echovirus 30. The last 3 samples were negative for VP1 PCR and were identified as Coxsackievirus A4 by 5′UTR PCR and sequencing. Based on the partial VP1 gene and 5′UTR region, the homologous comparison results for the isolates from the same serotypes were 92.5–99.4% (nucleotide) (Table S1).

**Figure 2:**
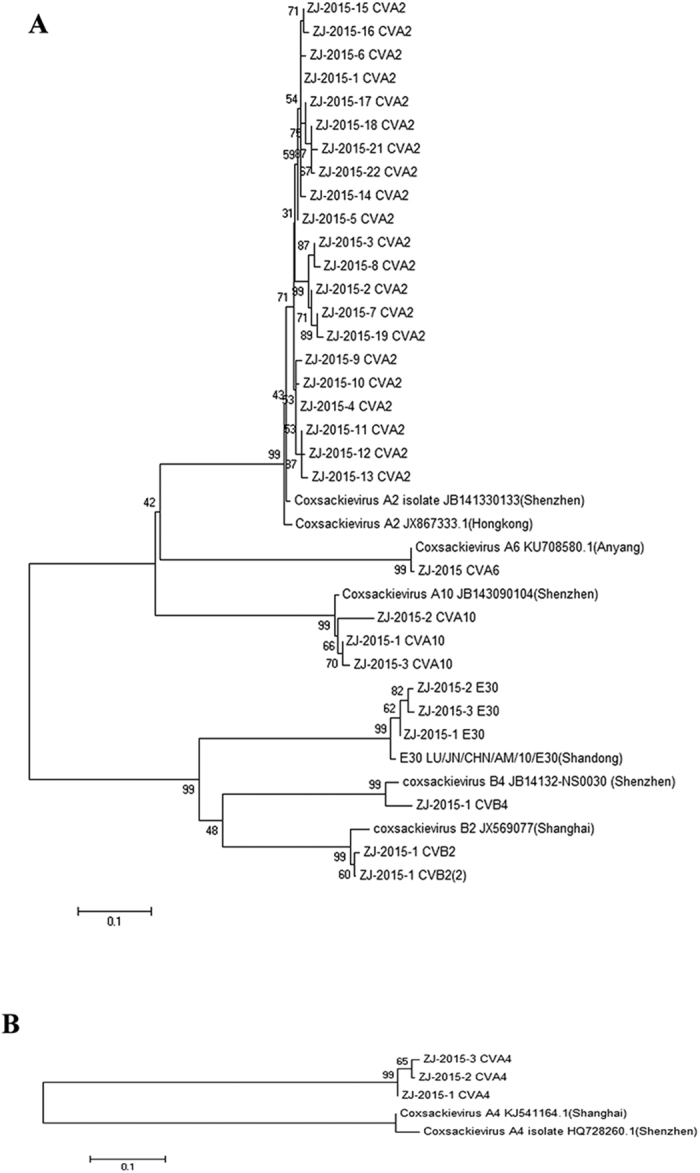
Phylogenetic analysis of the enterovirus isolates based on the partial VP1 gene or 5′UTR sequences. (**A**) Phylogenetic analysis of the enterovirus isolates based on the partial VP1 gene sequences; (**B**) Phylogenetic analysis of the enterovirus isolates based on the partial 5′UTR sequences.

Based on the VP1 gene and 5′UTR region sequences, phylogenetic analysis for the EVs of this study was done by comparison with all available VP1 gene sequences from the Genbank. From the constructed phylogenetic tree (Fig. 2), we can find all clinical isolates from Zhejiang belonged to the human EVA and EVB species. The CVA2, CVA10 and CVB4 isolates were most closely related to the stains from Shenzhen (Southern China). CVB2 isolate was most closely related to the stains from Shanghai (Southern China) and E30 isolates were most closely related to the stains from Shandong (Northern China).

## Discussion

Herpangina, associated with various enterovirus serotypes, is a commonly prevalent illness in young children^13^,^14^. In the summer of 2015, large outbreak of herpangina happened in Hangzhou, China. From May to August, 10 210 children were diagnosed with herpangina with the highest prevalence rate in June. Among the infected children, 91.6% were less than 5 years with high rate in 1–3 years children. We also found that the level of CRP slightly increased in children with herpangina during the study period.

Enteroviruses infection occurred in summer throughout the world^15^. To confirm whether enteroviruses are the major pathogens causing herpangina in Hangzhou, China, real-time RT-PCR targeting to highly conserved region in the human enteroviruses (HEV) genome was performed to detect the enterovirus. Among 2 310 throat swabs, 1 651(71.5%) were tested positive for enterovirus. The children aged 1 to 5 years old were the most susceptible population with a peak incidence in 1–3 years, which was similar to all herpangina aging incidence. Most importantly, children older than 3 years also exhibited high infection rate in Hangzhou. Amongpatients belonged to kindergarten and primary school age, cross infections occurred. To control infection, a short-term suspension was conducted in someschools.

As reported in a previous study, herpangina was associated with different strains of enteroviruses, such as CVA2 in Taipei in 2008^16^, CVA5 in Korea in 2009^10^, CVA6 and CVA10 in France in 2010^17^, Coxsackievirus A8 in Thailand in 2012^11^, Coxsackievirus A9 in Brazilian Amazon in 2014^12^ and in Japan during 2000–2005. There are also some reports about enterovirus infections caused by CVA5, CVA6 and CVA10^18^. Our study has established CVA2 as the most possible prevalent cause of herpangina in Hangzhou in 2015. Our study investigated distinct clusters of CVA2, CVA4, CVA10 and E30 strains in relation to their geographic origins. Phylogenetic analysis showed that the Thai CVA2 strains were closely related to strains isolated from Shenzhen and Hongkong, China, in 2012. Most importantly, CVA2 was reported caused 2 children death with respiratory symptoms in Hongkong^19^. In our surveillance, we didn’t find death cases among children for diagnosis of herpangina. Besides surveillance of herpangina, our hospital has also conducted the surveillance of enterovirus-associated encephalitis. Different from herpangina, we found E30 was the major pathogen causing enterovirus-associated encephalitis and none of CVA2 samples were found (data not shown). These results indicated that E30 may more easily invade to brain system of children.

This is the first report about the large outbreak of enterovirus herpangina among children in hangzhou, China. And the associated major subtype of enterovirus may be HEV A with high incidence of CVA2. In further studies, we will sequence more clinical enterovirus samples from EV-positive children with herpangina which would be helpful to further surveillance of enterovirus.

## Materials and Methods

### Patients and definitions

The Children’s Hospital of Zhejiang University School of Medicine is the largest comprehensive center for pediatric health care in Zhejiang province. This was a retrospective study conducted from May 2015 to August 2015 in the hospital. Patients who met the following criteria were enrolled: (1) age less than 14 years old, (2) all children who visit our hospital in the study period, (3) all children who were diagnosed with herpangina (well-characterized multiple vesicular exanthema and ulcers of the soft palate with presentation of fever, sore throat and anorexia) in our hospital in the study period.

### Ethical approval and informed consent

This retrospective study and method were approved by the medical ethics committee of the Children’s Hospital of Zhejiang University School of Medicine (NO. 2015-PRIB008) and all experiments were performed in accordance with relevant guidelines and regulations. Informed consent was obtained from all subjects.

### Routine blood test and C-reactive protein

White blood cell (WBC) counting, the proportion of lymphocyte and neutrophils were measured by Sysmex blood cell instrument (Sysmex 800i, Janpan). Concentrations of C-reactive protein (CRP) were measured by the QuikRead go instrument with QuikRead go CRP kits (Orion Diagnostica, Finland).

### Detection of enterovirus

Throat swabs were collected from children with the symptoms of herpangina. RNA was extracted by Nucleic acid automatic extraction instrument (Zhi-jiang company, Shanghai, China). The detection of EV was performed in ABI Stepone plus system by commercial one-step real-time RT–PCR assay kit (Zhi-jiang company, Shanghai, China). The real time RT-PCR was conducted under these conditions: 15 min at 50 °C, 5 min at 95 °C, and then followed by 40 cycles of 15 sec at 94 °C and 45 sec at 55 °C. Samples with CT value less than 35.0 were identified positive.

### VP1 and 5′UTR gene sequencing and phylogenetic analysis

All primers used in VP1 gene amplification were based on previous study^20^. Reverse transcription PCR (RT-PCR) kit (Invitrogen, Shanghai, China) was used to perform enterovirus cDNA synthesis. The total volume per RT-PCR was 10 μL which included 2 μL buffer (5×), 0.4 μL dNTP (10 mM), 1 μL DTT, 0.2 μL primer mix (Zhi-jiang company, Shanghai, China), 0.5 μL Superscript III, 5 μL Enterovirus RNA, 0.4 μL RNasin. The reaction was under these conditions: 22 °C, 10 min; 45 °C, 45 min; 95 °C, 5 min. The first round PCR of the VP1 gene was carried out in a mixture with a total volume of 50 μL that included 10 μL of the RT-PCR product, 0.5 μL DSC Taq (Enzymatics), 2.5 μL outer primers (Zhi-jiang company, Shanghai, China). The amplification was under the following conditions: 95 °C, 5 min; 40 cycles×(95 °C, 30 s; 42 °C, 30 s; 60 °C, 45 s); 72 °C, 10 min. The second round PCR was carried out in a mixture with a total volume of 50 μL that included 1 μL of the first round PCR product, 0.5 μL DSC Taq (Enzymatics), 2.5 μL outer primers (Zhi-jiang company, Shanghai, China). The amplification was under following conditions: 95 °C, 6 min; 40 cycles×(95 °C, 30 s; 60 °C, 20 s; 72 °C, 25 s); 72 °C, 10 min. The PCR product was sequencing in Majorbio (Shanghai, China). For VP1 PCR negative samples, we used 5′UTR primers (Zhi-jiang company, Shanghai, China) to amplificate and sequence 5′UTR region. The protocol is the same to VP1 PCR. VP1 gene and 5′UTR DNA sequences of the EV isolated were contrasted with the National Center for Biotechnology Information (NCBI) database through BLAST. Based on the sequences of the VP1gene, phylogenetic analysis was done by using the Mega 5.1software. The tree was constructed by using the neighbor-joining method. Significance of phylogenies was investigated by bootstrap analysis with 1,000 pseudoreplicate data sets. Bootstrap values of are indicated on the tree.

## Additional Information

**How to cite this article**: Li, W. *et al*. Large outbreak of herpangina in children caused by enterovirus in summer of 2015 in Hangzhou, China. *Sci. Rep.* **6**, 35388; doi: 10.1038/srep35388 (2016).

## Supplementary Material

Supplementary Information

## Figures and Tables

**Table 1:** Routine tests of children with herpangina.

Age	white blood cells	Lymphocyte(%)	Neutrophil(%)	CRP(mg/L)
**<3 years**	7.4 × 10^9^/L	46.4%	58.5%	25.1
**3–5 years**	8.9 × 10^9^/L	38.2%	54.3%	15.3
**>5 years**	8.2 × 10^9^/L	30.3%	63.2%	14.8
