# Supplementary material for: Large outbreak of herpangina in children caused by enterovirus in summer of 2015 in Hangzhou, China
Source: Sci Rep. 2016 Oct 18;6:35388. doi: 10.1038/srep35388 (PMC5067559; doi:10.1038/srep35388)
Supplement: Supplementary Information [file srep35388-s1.doc]

**Title:** Large outbreak of herpangina in children caused by enterovirus in summer of 2015 in Hangzhou,China

Wei Li, Hui-hui Gao, Qiong Zhang, Yu-jie Liu, Ran Tao, Yu-ping Cheng, Qiang Shu & Shi-qiang Shang


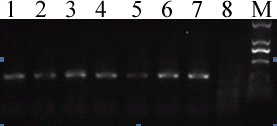


**Figure.S1** Enteroviruses VP1 gene PCR amplification.

1-7: product of amplification 8: negative control M: marker DL2000

| Serotype | No. of isolates | Reference strains | Length of product (bp) | Comparison between isolates  nt (%) | Comparison with reference strains  nt (%) |
| --- | --- | --- | --- | --- | --- |
| A2 | 22 | Fleetwood | 363 (VP1) | 95.3~98.0 | 94.1~98.4 |
| A4 | 3 | HighPoint | 314 (5UTR) | 92.5~96.4 | 90.9~95.4 |
| A6 | 1 | Gdula | 322（VP1） | NA | 95.8% |
| A10 | 3 | Kowalik | 359 (VP1) | 96.5~99.4 | 91.7~98.1 |
| B2 | 2 | Ohio-1 | 387 (VP1) | 98.3 | 96.5-97.2 |
| B4 | 1 | JVB | 381 (VP1) | NA | 97.9 |
| E30 | 3 | Bastianni | 386 (VP1) | 96.5~98.2 | 97.2~99.5 |

**Table.S1** Sequence analysis result of the enterovirus isolates.
